# Maternal Prenatal Positive Affect, Depressive and Anxiety Symptoms and Birth Outcomes: The PREDO Study

**DOI:** 10.1371/journal.pone.0150058

**Published:** 2016-02-26

**Authors:** Anu-Katriina Pesonen, Marius Lahti, Tiina Kuusinen, Soile Tuovinen, Pia Villa, Esa Hämäläinen, Hannele Laivuori, Eero Kajantie, Katri Räikkönen

**Affiliations:** 1 Institute of Behavioral Sciences, University of Helsinki, Helsinki, Finland; 2 Department of Obstetrics and Gynecology, University of Helsinki, Helsinki, Finland; 3 Department of Clinical Chemistry, University of Helsinki, Helsinki, Finland; 4 HUSLAB, Helsinki University Central Hospital, Helsinki, Finland; 5 Medical Genetics, University of Helsinki and Helsinki University Hospital, Helsinki, Finland; 6 Obstetrics and Gynecology, University of Helsinki and Helsinki University Hospital, Helsinki, Finland; 7 Institute for Molecular Medicine Finland, University of Helsinki, Helsinki, Finland; 8 National Institute for Health and Welfare, Helsinki, Finland; 9 Children's Hospital, Helsinki University Central Hospital and University of Helsinki, Helsinki, Finland; 10 Department of Obstetrics and Gynecology, Oulu University Hospital and University of Oulu, Oulu, Finland; Chiba University Center for Forensic Mental Health, JAPAN

## Abstract

**Background:**

We investigated whether maternal prenatal emotions are associated with gestational length and birth weight in the large PREDO Study with multiple measurement points of emotions during gestation.

**Methods:**

Altogether 3376 pregnant women self-assessed their positive affect (PA, Positive and Negative Affect Schedule) and depressive (Center for Epidemiologic Studies Depression Scale, CES-D) and anxiety (Spielberger State Anxiety Scale, STAI) symptoms up to 14 times during gestation. Birth characteristics were derived from the National Birth Register and from medical records.

**Results:**

One standard deviation (SD) unit higher PA during the third pregnancy trimester was associated with a 0.05 SD unit longer gestational length, whereas one SD unit higher CES-D and STAI scores during the third trimester were associated with 0.04–0.05 SD unit shorter gestational lengths (P-values ≤ 0.02), corresponding to only 0.1–0.2% of the variation in gestational length. Higher PA during the third trimester was associated with a significantly decreased risk for preterm (< 37 weeks) delivery (for each SD unit higher positive affect, odds ratio was 0.8-fold (P = 0.02). Mothers with preterm delivery showed a decline in PA and an increase in CES-D and STAI during eight weeks prior to delivery. Post-term birth (≥ 42 weeks), birth weight and fetal growth were not associated with maternal prenatal emotions.

**Conclusions:**

This study with 14 measurements of maternal emotions during pregnancy show modest effects of prenatal emotions during the third pregnancy trimester, particularly in the weeks close to delivery, on gestational length. From the clinical perspective, the effects were negligible. No associations were detected between prenatal emotions and birth weight.

## Introduction

Mounting empirical evidence suggests that maternal emotional distress during pregnancy, including depressive symptoms, and state, trait and pregnancy-specific anxiety, may increase the risk of adverse birth outcomes, such as preterm birth (< 37 0/7 gestational weeks) or shorter length of gestation, intrauterine growth restriction (IUGR), and low (< 2.5 kg) or lower birth weight at delivery [1–7]. However, the literature is relatively inconsistent, and includes null associations as well [8]. The associations may also vary according to continuous or categorical treatment of the exposure and/or outcome variables, and the level of income in the country in which the sample was derived [1,4,5,7].

A small body of literature suggests that maternal positive predisposition or positive attitude towards pregnancy may, independently of emotional distress, decrease the risk of adverse birth outcomes. In a sample of 130 pregnant women, higher levels of self-esteem and optimism measured in gestational weeks 28–30 were associated with higher birth weight, but not with length of gestation [9]. Correspondingly, a higher level of dispositional optimism measured in gestational weeks 21–30 was associated with higher birth weight, but not with length of gestation [10], among medically high-risk sample of 129 pregnant women. A more recent study in 169 pregnant women demonstrated that more positive attitudes towards pregnancy, measured on average at gestational week 15, were associated with a longer length of gestation and a lower risk of preterm delivery. This study additionally showed that a steeper increase in maternal positive attitudes towards gestational weeks 19 and 30 was associated with a longer gestational length, but it was not associated with prematurity [11]. While these studies provide important insight into the potentially beneficial effects of positive characteristics, all of the existing studies have been conducted in relatively small and/or selected samples and none have covered the whole pregnancy. This has precluded testing whether the effects would vary according to the timing of the exposure. In addition, the measurement of positive affect has mostly referred to attitudes towards pregnancy or dispositional trait-like measures of the pregnant mother, and less to state-like fluctuations in current affect.

Our original contribution in this study is to explore the influence of positive affect and the role of the timing of exposures for the birth outcomes. To our knowledge, our study offers the most comprehensive view on maternal emotional state over the course of pregnancy reported thus far, with prospective assessments of emotions at two-week intervals from 12 weeks’ gestation onwards in a large data set. Therefore, the roles of negative affect and its timing were also relevant. Hence, we examined the hypothesis that higher positive and lower negative affect would predict longer length of gestation and higher birth weight in 3376 pregnant women. We also examined whether the associations would vary by the medical risk-status of mothers, since obstetric complications are associated with higher emotional distress during pregnancy [12].

## Methods

### Participants

The participants came from the Prediction and Prevention of Preeclampsia (PREDO) Study [13–15]. The PREDO is composed of two subsamples. For the high-risk subsample, we recruited 973 singleton pregnant women with risk factors for preeclampsia and 110 singleton pregnant women without known risk factors as a comparison group at 12 0/7–13 6/7 gestational weeks as previously described [13,15]. For the community-based subsample, we recruited 3702 singleton pregnant women within the same pregnancy weeks. The recruitment took place between September 2005 and February 2010 when these women attended their first ultrasound scan in one of ten hospital maternity clinics participating in the PREDO. Of the 4785 recruited mothers, we had data on birth outcomes from the birth register and the two-week interval self-report of positive affect, depressive symptoms, and state anxiety during pregnancy for 3376 mothers (70.6% of those recruited). Participants of the study did not differ from mothers with missing data on prenatal emotions in subsample status or birth outcome (P-values > 0.29), but were 0.9 year older (95% Confidence Interval (CI) 0.6 to 1.2, P < 0.001), more often primiparous (40.7% vs. 33.9%, P < 0.001) and less often obese before pregnancy (body mass index (BMI) > 30 kg/m^2^) (13.3% vs. 17.1%, P = 0.002). The participants also less often smoked during pregnancy (3.3% vs. 9.3%, P < 0.001) and more often had tertiary education (59.9% vs. 46.4%, P < 0.001). The characteristics of the participants are presented in Table 1. The study protocol was approved by the Ethical Committee of Helsinki University Central Hospital, and all participants signed an informed consent.

**Table 1:** Characteristics of the participants of the Predo-Study.

Continuous variables		N	Mean/N	SD/%	Range
PANAS	1^st^ trimester	3200	30.3	7.9	10.0–50.0
	2^nd^ trimester	3357	30.2	7.0	10.0–50.0
	3^rd^ trimester	3240	30.3	7.8	10.0–50.0
CES-D	1^st^ trimester	3205	11.5	7.9	0.0–50.0
	2^nd^ trimester	3357	11.1	6.5	0.0–48.7
	3^rd^ trimester	3240	11.8	7.1	0.0–48.5
STAI	1^st^ trimester	3198	31.8	9.8	20.0–80.0
	2^nd^ trimester	3357	33.2	7.8	20.0–78.0
	3^rd^ trimester	3240	34.1	8.7	20.0–75.2
Social Support	1^st^ trimester	3216	47.0	13.3	1.0–65.0
	2^nd^ trimester	3359	42.5	12.1	0.0–65.0
	3^rd^ trimester	3240	43.3	13.2	0.0–65.0
Birth weight (grams)		3363	3526.7	518.5	580–5490
Gestational length (weeks)		3376	39.9	1.6	27.7–42.7
Mother's age at delivery (years)		3376	31.8	4.7	17.0–47.4
**Categorical variables**					
Birth outcome	SGA	3363	80	2.4	
	LGA	3363	69	2.1	
	Premature	3376	130	3.9	
	Postterm	3376	186	5.5	
Elective Cesarean section (yes)		3341	206	6.2	
Parity		3376			
	Primiparous		1369	40.7	
	Multiparous		1997	59.3	
Child's sex		3376			
	Boy		1741	51.6	
	Girl		1635	48.4	
Mother's education		3371			
	Basic		91	2.7	
	Secondary		1258	37.3	
	Tertiary		2022	60.0	
Mother’s prepregnancy BMI		3374			
	< 18.5		112	3.3	
	18.5–24.99		2180	64.6	
	25–29.99		641	19.0	
	>30		441	13.1	
Alcohol consumption during pregnancy		3336			
	No		2802	84.0	
	Yes		534	16.0	
Smoking during pregnancy		3374			
	No		3149	93.3	
	During 1^st^ trimester		113	3.3	
	During and after 1^st^ trimester		112	3.3	
Hypertensive pregnancy disorders		3374			
	Normotension		2968	88.0	
	Gestational hypertension		142	4.2	
	Any preeclampsia		125	3.7	
	Chronic hypertension		139	4.1	
Gestational diabetes		3374			
	No		3021	89.5	
	Yes		353	10.5	
Type I diabetes		3374			
	No		3355	99.4	
	Yes		19	0.6	
Antidepressant medication		2811			
	No		2734	97.3	
	Yes		77	2.7	
Other psychotropic medications		2811			
	No		2791	99.3	
	Yes		20	0.7	

PANAS, Positive and Negative Affect Schedule (only positive affect included); CES-D, Center for Epidemiologic Studies Depression Scale; STAI, Spielberger State Anxiety Scale; SGA, Small for gestational age birth weight < -2 SD according to Finnish growth charts; LGA, Large for gestational age birth weight > 2 SD according to Finnish growth charts; Preterm birth, birth < 37 0/7 weeks of gestation; Post-term birth, birth ≥ 42 0/7 weeks of gestation; BMI, body mass index.

### Measurement of variables

#### Prenatal emotions

The participants filled in well-validated questionnaires [16–18] on prenatal emotions at two week intervals throughout pregnancy from 120/7 to 13 6/7 gestational weeks until delivery or until 38 0/7 to 39 6/7 gestational weeks. The questionnaires were filled in up to 14 times during pregnancy.

Positive affect: We used Positive Affect (PA) scale comprising 10 mood states derived from the International Positive and Negative Affect Schedule (PANAS) [18]. The participants were asked to rate the extent to which they currently feel each mood state on a scale from 1 (not at all) to 5 (very much).

Depressive symptoms: We used the Center for Epidemiologic Studies Depression Scale (CES-D) (16) for both continuous and categorical variables: CES-D scores were dichotomized at ≥16, which is the cut-off for individuals at risk for clinically significant depressive symptoms [16]. The participants were asked to rate the 20 questions covering the frequency of symptoms experienced during the preceding week on a scale of 0 (not at all, less than one day) to 3 (all the time / 5–7 days).

Anxiety symptoms: To measure state anxiety, we used the Spielberger State Anxiety Scale (STAI) [17].The participants were asked to rate the 20 items covering the extent to which they currently feel anxious on a scale from 1 (not at all) to 4 (very much so).

#### Birth outcomes

Data on gestational length and birth weight came from the hospital birth records for the high-risk subsample and from the national birth register for the community-based subsample. We used the birth outcome variables as both continuous and categorical variables: preterm birth: ≤ 36 6/7 gestational weeks; term birth: 37 0/7-41 6/7 gestational weeks; post-term birth: ≥ 420/7gestational weeks; small for gestational age (SGA): birth weight for gestational age ≤ -2 standard deviations (SD); appropriate for gestational age (AGA): birth weight for gestational age > -2 SD—≤ 2SD; LGA: birth weight for gestational age ≥ 2 SDs, all according to Finnish national growth charts [19].

#### Confounding variables

Maternal age (years), delivery mode (vaginal vs. Cesarean), parity (primiparous vs. multiparous), smoking (no/quit during first trimester/smoked throughout pregnancy) during pregnancy, and infant’s sex (girl vs. boy) were derived from the national birth register for both subsamples. For the medical high-risk sample, maternal pre-pregnancy BMI (underweight: BMI < 18.5 kg/m^2^; normal weight: BMI = 18.5 to 25 kg/m^2^; overweight; BMI = 25 to 30 kg/m^2^; obese: BMI ≥ 30 kg/m^2^, hypertensive pregnancy disorders (preeclampsia, gestational hypertension or chronic hypertension), gestational diabetes (hyperglycemia that first emerged or was first identified during pregnancy), and Type I diabetes were derived from patient case records and verified by a clinical jury. For the community-based subsample the data of pregnancy disorders and BMI came from the national birth register. In both subsamples maternal education (primary: < 10 years; secondary: 10–12 years; tertiary: > 12 years) prenatal use of antidepressant or other psychotropic medication (sedatives, barbiturates and anti-psychotics) and alcohol consumption during pregnancy (yes/no) were self-reported in a questionnaire given at the first ultrasound screening at gestational week 12 6/7-13 6/7. Social support was assessed in at two-week intervals (i.e. biweekly) in the questionnaire with a 65 mm long visual analog scale.

### Statistical analyses

First, we used linear regression analyses to study associations between maternal positive affect, depressive symptoms and state anxiety with birth outcomes as continuous variables. Trimester-specific prenatal emotions were tested in separate models. CES-D and STAI scores were square-root- and logarithm-transformed to attain normality. All continuous variables were standardized to the mean of 0 and SD of 1 to facilitate interpretation of effect sizes. Hence, the unstandardized regression coefficients represent SD unit change per SD unit change. We calculated trimester mean scores of prenatal emotions (first trimester week 12 measurement; second trimester means of scores during weeks 14–26; third trimester week 28 to delivery or to week 38). The rationale of using trimester means in these analyses instead of the bi-weekly scores was based on the very high inter-correlations between the bi-weekly emotion scores (r range 0.41–0.80, all P-values < 0.001).

All analyses were adjusted for maternal age at delivery, parity, delivery mode, education and infant’s sex (Model 1) and further for maternal alcohol consumption and smoking during pregnancy, maternal pre-pregnancy body mass index, hypertensive pregnancy disorders, gestational and Type I diabetes, antidepressant and other psychotropic medication, and social support (Model 2). In case of significant associations, the explained variance (R^2^) was calculated for prenatal emotions by hierarchical multiple regression analyses. We used logistic regressions to examine whether maternal positive affect decreased and depressive symptoms and state anxiety increased the risk of preterm vs. term, post-term vs. term, SGA vs. AGA, and LGA vs. AGA births. Finally, to test whether the associations varied by the medical risk status of the sample, we added an interaction term ‘high-risk subsample vs. community subsample x trimester-specific PA/CES-D/STAI’ to the regression equations following the main effects. In these interaction analyses, the small comparison group recruited for the high-risk subsample was merged to the community-based subsample.

## Results

PA (range of Pearson r’s 0.57 to 0.83), CES-D (r’s 0.61 to 0.83) and STAI (r’s 0.55 to 0.79) scores were significantly correlated across the three trimesters (all P-values < 0.001). PA correlated negatively within the trimesters with STAI (r’s -0.51 to -0.66) and CES-D (r’s -0.50 to -0.62). STAI and CES-D correlated positively with each other within the trimesters (r’s 0.69 to 0.84), all P-values < 0.001. In terms of mean raw scores shown in Table 1, CES-D and STAI scores were significantly higher during the first and third trimesters compared to the second trimester (all P-values < 0.001), and significantly higher during the third trimester than during the first trimester (P-values ≤ 0.002). No mean score differences were found for PANAS (all P-values ≥ 0.26).

### Birth outcomes as continuous

Table 2 shows the trimester-specific associations between PA, CES-D and STAI and birth outcomes. One SD unit increase in PA and one SD unit decrease in CES-D and STAI during the third trimester were associated with 0.05 SD unit longer and 0.04 and 0.05 SD unit shorter gestational length, respectively. These associations remained significant after we made adjustments for the additional confounders in Model 2. PA, CES-D, and STAI at the first and second trimesters were not associated with gestational length (P-values ≥ 0.17), and PA, CES-D, and STAI at any trimester were not associated with birth weight (P-values ≥ 0.16). In hierarchical multiple regression analyses, independent background variables (maternal age at delivery, parity, delivery mode, education, infant’s sex, maternal alcohol consumption and smoking during pregnancy, maternal pre-pregnancy BMI, hypertensive pregnancy disorders, gestational and Type I diabetes, antidepressant and other psychotropic medication, and social support) explained 8.7% of the variance in gestational length, whereas PA, CES-D or STAI during the third trimester explained only 0.2%; 0.1% and 0.2% of the variance, respectively (P-values ≤ 0.02 for the change).

**Table 2:** The associations between prenatal positive affect, depressive and anxiety symptoms with birth outcome.

	Gestational weeks SD				Birth weight SD			
	Model 1		Model 2		Model 1		Model 2	
	B	95% CI	B	95% CI	B	95% CI	B	95% CI
**PANAS**								
1^st^ trimester	0.01	-0.03;0.04	0.01	-0.03;0.04	-0.00	-0.04;0.03	-0.01	-0.04;0.03
2^nd^ trimester	0.01	-0.02;0.04	0.01	-0.03;0.04	0.00	-0.03;0.03	-0.00	-0.03;0.04
3^rd^ trimester	0.05**	0.01;0.08	0.05*	0.01;0.08	-0.01	-0.04;0.02	-0.00	-0.04;0.03
**CES-D**								
1^st^ trimester	-0.01	-0.04;0.03	-0.00	-0.04;0.03	0.02	-0.02;0.05	0.02	-0.02;0.05
2^nd^ trimester	-0.02	-0.05;0.02	-0.02	-0.06;0.01	0.01	-0.02;0.05	0.01	-0.03;0.04
3^rd^ trimester	-0.04*	-.007;-0.00	-0.04*	-0.07;-0.00	0.02	-0.01;0.06	0.01	-0.02;0.05
**STAI**								
1^st^ trimester	-0.02	-0.06;0.01	-0.03	-0.06;0.01	-0.01	-0.04;0.02	-0.01	-0.05;0.02
2^nd^ trimester	-0.02	-0.05;0.01	-0.02	-0.05;0.02	0.00	-0.03;0.04	0.00	-0.04;0.04
3^rd^ trimester	-0.05**	-0.08;-0.02	-0.05*	-0.08;-0.01	0.02	-0.02;0.05	0.01	-0.03;0.04

**Model 1** was adjusted for child’s sex, parity, elective Cesarean section, and mother’s age at delivery and education; **Model 2** was adjusted additionally for prenatal alcohol use and smoking, mother’s pre-pregnancy BMI, hypertensive pregnancy disorders, gestational and Type I diabetes, antidepressant and other psychotropic medication, and social support.

PANAS, Positive and Negative Affect Schedule (only positive affect included); CES-D, Center for Epidemiologic Studies Depression Scale; STAI, Spielberger State Anxiety Scale.

*p ≤ 0.05

**p ≤ 0.01

### Birth outcomes as categorical

PA during the third trimester was associated with a significantly decreased odds for preterm delivery (for each SD unit increase in PA, the Odds Ratio (OR) decreased by 0.8-fold (95% CI 0.7; 1.0, P = 0.016 in Model 1; OR = 0.8, 95% CI 0.6; 1.0, P = 0.025 in Model 2) (these analyses excluded two infants born before the third trimester). CES-D (OR = 1.2, 95% CI 1.0–1.4, P = 0.066 in Model 1, P = 0.12 in Model 2), and STAI (OR 1.2, 95% CI 1.0–1.4, P = 0.091 in Model 1; P = 0.26 in Model 2) scores during the third trimester, in turn, were higher in women with a preterm delivery, but these associations were not statistically significant. PA, CES-D, and STAI at the first and second trimesters were not associated with a preterm delivery (P-values ≥ 0.44) and PA, CES-D, and STAI at any trimester were not associated with a post-term delivery (P-values ≥ 0.20) or with SGA vs. AGA, or LGA vs. AGA status of the infant (P-values ≥ 0.087).

**Fig 1:**
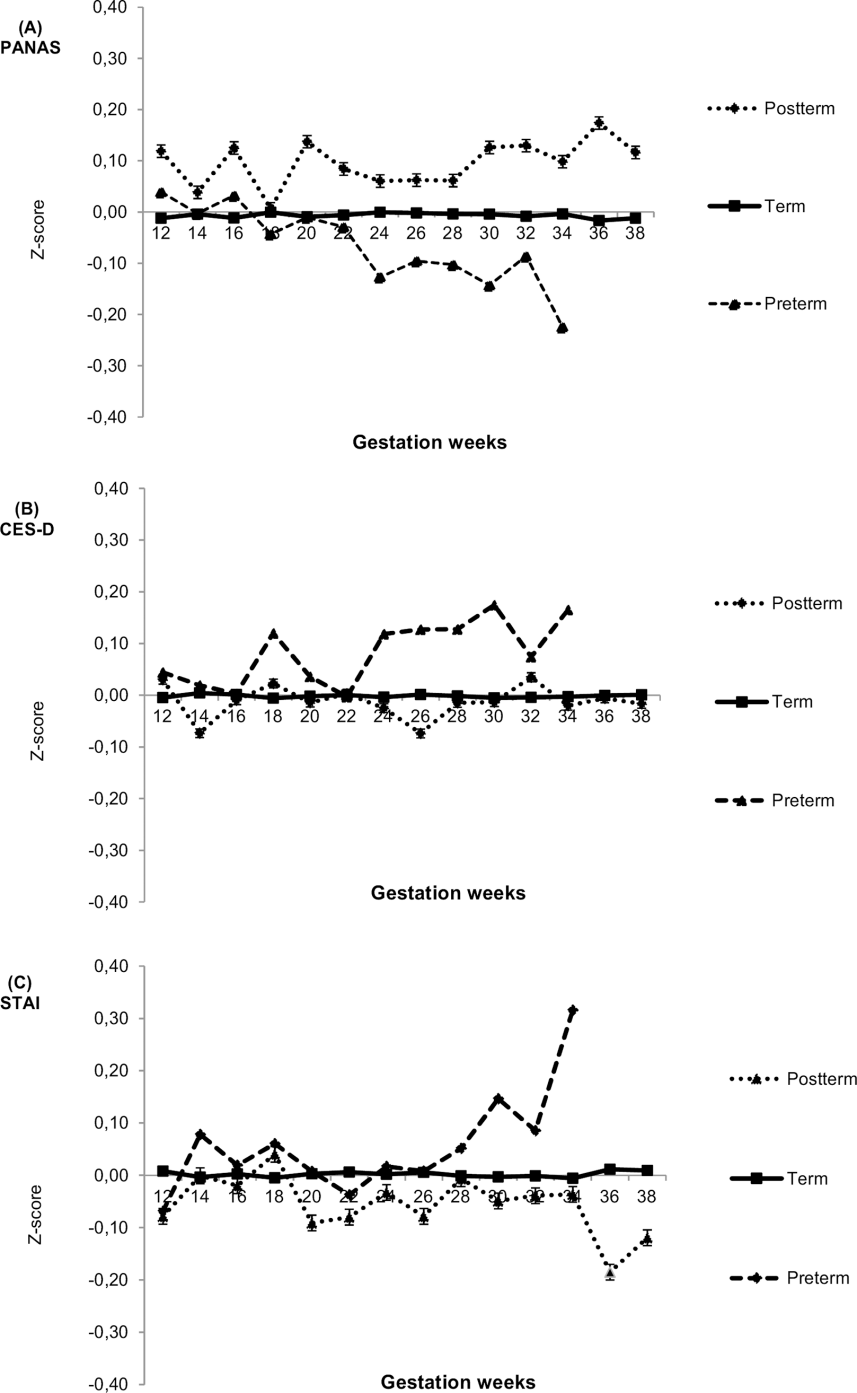
Trajectories of maternal (A) positive affect (PANAS), (B) depressive symptoms (CES-D), and (C) anxiety symptoms (STAI) at two-week intervals according to preterm (≤ 36 6/7 gestational weeks), term (37 0/7-41 6/7 gestational weeks) and post-term delivery (≥ 42 0/7 gestational weeks).

Post-hoc exploratory analyses further highlighted the significance of the trajectories of emotions experienced during pregnancy. Fig 1 shows the trajectories of standardized values of prenatal emotions in three groups divided by the gestational length (preterm, term, post-term). In comparison with mothers with a term delivery, mothers with a preterm delivery showed a decrease in PA (P = 0.002 for the difference between mean prenatal emotion score during the last eight weeks of pregnancy and the mean score derived from earlier measurement points during pregnancy) and an increase in both CES-D (P = 0.038) and STAI (P = 0.037) scores towards the end of pregnancy.

### Moderation by medical risk status of the sample

Medical risk status moderated significantly the associations between the third-trimester CES-D and gestational length (P for high-risk vs community sample x CES-D interaction = 0.012 in Model 1, P = 0.032 in Model 2, respectively). In the high-risk sample, one SD unit increase in the third trimester CES-D was associated with a 0.12 SD unit shorter gestational length (95% CI -0.19 to -0.04, P = 0.003 in Model 1; P = 0.003 in Model 2); in the community sample this association was not significant (P-values > 0.28).

## Discussion

We investigated among 3376 women whether prenatal PA and depressive and anxiety symptoms were associated with the likelihood of shorter gestation, preterm birth or lower birth weight. While the issue has been widely studied, the previous literature concentrates mostly on negative emotions and does not cover the effect of the emotions experienced throughout the entire pregnancy. With our data, we were able to overcome these issues with up to 14 measurement points during pregnancy, allowing a trimester-specific approach, with an adjustment for several major confounders. We found statistically significant, but clinically negligible associations between positive and negative emotions during the third trimester and gestational length, and no significant associations with birth weight. In addition, PA during the third trimester was associated with lower odds for a preterm birth.

PA was associated with longer gestational length and negative emotions with shorter gestational length. The general pattern of the results was in accordance with earlier studies [2–7], which have not, however, provided trimester-specific information with multiple measurement points, as done here. We found high correlations between the bi-weekly measurements of emotions. Although this may suggest that measurement of emotions once in each trimester would be sufficient for future studies, we would also like to point out that with increasing measurement points, there is a decrease in measurement error, resulting in more reliable estimates of prenatal emotions in each trimester. The observed associations held when the models were adjusted for an extensive list of covariates. However, prenatal emotions independently explained only 0.1–0.2% of the variation in gestational length, which is an extremely modest amount. This effect corresponds to 12 hours’ shorter or longer gestation for each SD unit change in prenatal emotions.

With regard to prematurity, higher PA during the third trimester was associated with a reduced risk for a premature delivery, in line with a recent study [11]. However, they assessed positive emotions only three times during the pregnancy. We also found a non-significant trend for a higher risk of prematurity in those scoring higher on CES-D and STAI during the third trimester, but these associations clearly declined in the fully adjusted models. If the mean value of depressive symptoms was ≥ 16 points in CES-D in the third trimester, the risk for a premature birth was 1.5-fold, but this association was not significant in either model. However, the magnitude of this effect is in accordance with two meta-analyses on the relation between prenatal depression and increased risk for prematurity [4,5]. Similar odds ratios were also reported in the meta-analysis on the effects of prenatal anxiety on the risk of preterm birth [2]. Importantly, when we examined the prenatal affect during eight weeks prior to the delivery, we found a significant decrease in PA and an increase in depressive and anxiety symptoms among mothers with a preterm delivery, relative to mothers with term deliveries, again highlighting the modest links between gestational length and prenatal emotions, particularly in the later stages of gestation. In contrast, emotions during the first and second trimesters were unrelated to gestational length.

Kramer [6] presented a large variety of different measures of acute and chronic stressors and psychological distress to mothers in the second trimester and reported that of all forms of stress and psychological distress, only pregnancy-specific anxiety was associated with spontaneous preterm delivery, a finding that has been described before [10,20,21]. The authors speculated that this result may also indicate a reversed causality since pregnancy-specific anxiety is also a mother’s perception of potential medical risks. However, these concepts may interact; pregnancy-specific and general anxiety influence each other over time, resulting in accumulating anxiety for some mothers during pregnancy [22].

We did not observe any associations of PA, prenatal anxiety, or depression with birth weight. These findings are in line with a meta-analysis that noted no associations between depression and birth weight [4] and with a study [8] focusing only on mothers with diagnosed depressive/or anxiety disorders, and observing no associations with birth weight. However, our findings are in contrast to other meta-analytic studies showing that maternal depression [5] and anxiety [2] during pregnancy predict an increased risk of low birth weight, to the Generation R Study reporting an association between anxiety symptoms at the second trimester and lower birth weight [23], and to studies showing that positive dispositions may be associated with larger birth weight [9,10].

The current study subject has been under active research, with several biological mechanisms potentially underlying associations between prenatal emotions and gestational length. Increased concentration of corticotrophin-releasing hormone (CRH) in early pregnancy plasma predicts preterm birth [24–26]. Most of the CRH during pregnancy is produced by the placenta; this production is stimulated by circulating cortisol [27–29]. With CRH stimulating cortisol secretion by the placenta and the fetal adrenal cortex, this has been suggested to create a positive feedback loop ultimately leading to delivery [27–29]. However, the evidence for the associations between maternal depression during pregnancy and elevated CRH concentrations or cortisol levels is inconclusive including both positive [30–33] and null findings [6]. The placenta plays a central role in regulating transfer of glucocorticoids to the fetus: The placental enzyme 11-beta hydroxysteroid dehydrogenase type 2 (HSD2) metabolizes most of active maternal cortisol to inactive cortisone, protecting the fetus from cortisol overexposure [34]. In a subsample of term births in the current PREDO Study we quantified placental mRNA levels of glucocorticoid (GR) and mineralocorticoid (MR) serotonin receptor genes as well as levels of 11-beta HSD2 and 11-beta hydroxysteroid dehydrogenase type 1 (HSD1) [13,14]. We found that maternal antenatal depression was associated with increased placental mRNA expression of both MR and GR, thus increasing placental glucocorticoid sensitivity, and suggesting the existence of a mechanism for increased fetal glucocorticoid exposure following maternal antenatal distress [14]. The associations were strongest for the third trimester depression scores. We also reported an association between higher GR mRNA levels and shorter gestation within the term range [13]. These results suggest that maternal antenatal mood is associated with placental glucocorticoid function, by both regenerating active glucocorticoids in placenta and increasing sensitivity to glucocorticoids.

The strengths of this study include the large well-characterized sample and the multiple repeated measurements of PA and anxiety and depressive symptoms, enabling us to reliably evaluate the effects of these factors on gestation length and birth size and to assess specific sensitivity periods. We were also able to assess possible confounding by multiple factors known to affect gestation length and birth size.

As to limitations, we cannot rule out that the results, especially with regard to prematurity, reflect reverse causality, i.e. prenatal emotions would be then generated as a response to the risk of premature birth. Mothers in Finland are followed intensively during pregnancy, with mothers being well aware of potential risks. Second, PA had a strong inverse correlation with negative emotions. We cannot say whether PA had an independent effect on gestational length or whether the effect could be reduced by a lack of negative emotions (depressive and anxiety symptoms). This is, however, a semantic question related to the core definition of positive emotions and their relation to depression and anxiety. Third, we did not have information about whether mothers with depression and anxiety symptoms received any psychosocial support. Finally, the participants may not be representative of the catchment areas of the study hospitals; these mothers had on average high educational attainment and smoking during pregnancy was rare. This would be expected to cause bias only if the association between prenatal emotions and pregnancy outcomes differs between participants and non-participants.

In summary, we used a large sample of pregnant women who rated their prenatal emotions bi-weekly to examine the relationships of PA and depressive and anxiety symptoms with gestational length and birth weight. Experience of PA during the third trimester was associated with longer gestation and lower odds of preterm delivery, whereas higher prenatal depressive and anxiety symptoms during the third trimester were associated with shorter gestation. The associations regarding depressive symptoms and gestational length were stronger among high-risk mothers. We also found that the course of emotions during the last eight weeks of pregnancy is different among women delivering preterm, as they displayed more negative emotions and less PA. Birth weight and fetal growth were not associated with prenatal emotions, and no effects on gestational length were observed for the first two trimesters. Our findings thus highlight the modest effects of both positive and negative emotions during the third pregnancy trimester on gestational length. While this subject has raised intense research interest in recent years, our findings indicate that the effects of prenatal emotions on birth characteristics are very small.

The clinical relevance may be in the message that prenatal negative emotions, including symptoms of depression and anxiety, may not play such a large role for pregnancy outcome *per se*. Of course, this has to be verified in further studies with as intensive measurements of prenatal emotions.

However, several studies show that prenatal negative emotions may be harmful to child development, being associated with increased psychopathology in the offspring, often independently of the maternal postnatal emotions [35–38]. As maternal prenatal emotions are likely to persist to the postnatal period [39], there is however a risk for an accumulative risk for a less optimal child development through less adaptive parent-child interaction patterns. New evidence is also emerging on the links between maternal prenatal stress and emotions and epigenetic programming of genes regulating the HPA axis in the offspring [40,41]. We have shown that higher placental expression of genes regulating feto-placental glucocorticoid and serotonin exposure is likely to mediate partly the associations between higher maternal depressive symptoms during the third trimester of pregnancy and regulatory behavioral challenges of the infant [42]. Together with this evidence, our present findings suggest that the burden of maternal prenatal negative emotions to the child may be transferred otherwise than through the immediate birth outcome.
